# Supplementary material for: Lateralized hippocampal oscillations underlie distinct aspects of human spatial memory and navigation
Source: Nat Commun. 2018 Jun 21;9:2423. doi: 10.1038/s41467-018-04847-9 (PMC6013427; doi:10.1038/s41467-018-04847-9)
Supplement: Supplementary file 1 — Supplementary Information [file 41467_2018_4847_MOESM1_ESM.pdf]

Lateralized hippocampal oscillations underlie distinct aspects of human spatial memory and navigation

Jacobs et al.

Supplementary Information

Lateralized hippocampal oscillations underlie distinct aspects of human spatial memory and navigation

Jonathan Miller[1], Andrew J. Watrous[1], Melina Tsitsiklis[2], Sang Ah Lee[3], Sameer A. Sheth[4], Catherine A. Schevon[5], Elliot H. Smith[6], Michael R. Sperling[7], Ashwini Sharan[8], Ali Akbar Asadi-Pooya[7,9], Gregory A. Worrell[10], Stephen Meisenhelter[11], Cory S. Inman[12], Kathryn A. Davis[13], Bradley Lega[14], Paul A. Wanda[15], Sandhitsu R. Das[13], Joel M. Stein[16], Richard Gorniak[17], Joshua Jacobs*[1]

[1]Department of Biomedical Engineering, Columbia University, New York, NY
[2]Doctoral Program in Neurobiology and Behavior, Columbia University, New York, NY
[3]Department of Bio and Brain Engineering, Korea Advanced Institute of Science and Technology, Daejeon, Korea
[4]Department of Neurosurgery, Baylor College of Medicine, Houston, TX
[5]Department of Neurology, Columbia University Medical Center, New York, NY
[6]Department of Neurological Surgery, Columbia University Medical Center, New York, NY
[7]Department of Neurology, Thomas Jefferson University, Philadelphia, PA
[8]Department of Neurosurgery, Thomas Jefferson University, Philadelphia, PA
[9]Shiraz Neurosciences Research Center, Shiraz University of Medical Sciences, Shiraz, Iran
[10]Department of Neurology, Mayo Clinic, Rochester, MN
[11]Department of Neurology, Geisel School of Medicine at Dartmouth, Lebanon, NH
[12]Emory University School of Medicine, Atlanta, GA
[13]Department of Neurology, Hospital of the University of Pennsylvania, Philadelphia, PA
[14]University of Texas–Southwestern, Dallas, TX
[15]Department of Psychology, University of Pennsylvania, Philadelphia, PA
[16]Department of Radiology, Hospital of the University of Pennsylvania, Philadelphia, PA
[17]Department of Radiology, Thomas Jefferson University, Philadelphia, PA

*Correspondence: joshua.jacobs@columbia.edu, 351 Engineering Terrace, Mail Code 8904, 1210 Amsterdam Avenue, New York, NY 10027, 212-854-2445

Subject	Age	Gender	H/L	Site	Electrode Locations	Accuracy
1	30	M	R/L	D	L FC, PC	0.84
2	41	F	R/L	J	R FC, TC, MTL; L FC, TC, PC, MTL, H	0.84
3	25	M	R/U	C	R FC, OC, TC, PC, MTL, H; L OC, TC	0.72
4	46	M	R/U	J	R FC, TC, PC, MTL, H	0.60
5	48	M	R/L	P	L FC, OC, TC, PC, H	0.50
6	36	F	R/L	D	L FC, OC, TC, PC, H	0.79
7	37	M	R/L	D	R FC; L FC, PC	0.77
8	37	M	R/L	D	R FC; L FC, PC	0.79
9	28	M	R/L	D	R FC, TC, PC; L FC	0.79
10	22	M	R/L	C	R FC, OC, TC, PC, MTL, H	0.80
11	46	M	R/L	T	R TC, H; L FC, OC, TC, PC, MTL, H	0.68
12	33	M	R/L	M	L FC, OC, TC, PC, H	0.89
13	24	M	R/L	T	R FC, TC, PC, H; L FC, TC, MTL, H	0.79
14	21	M	R/U	J	L FC, OC, TC, PC	0.52
15	21	M	R/U	J	L FC, OC, TC, PC	0.50
16	36	M	R/U	M	R FC, TC, MTL, H; L TC, MTL, H	0.49
17	22	F	R/L	E	R FC, TC, MTL, H; L TC, H	0.47
18	21	F	R/L	C	L FC, TC, MTL, H	0.74
19	26	F	R/U	C	R TC, H	0.60
20	23	M	R/U	M	R FC, PC	0.81
21	58	F	R/L	P	R FC, TC, MTL; L FC, TC, PC, H	0.66
22	19	M	R/U	J	R FC, TC, PC, MTL, H	0.57
23	28	M	R/L	C	R FC; L FC, TC, H	0.67
24	24	F	R/U	E	R TC, MTL; L FC, OC, TC, PC, MTL, H	0.68
25	44	M	R/L	E	R FC, TC, PC; L FC, OC, TC, PC, H	0.82
26	37	M	R/L	P	R FC, OC, TC, PC, MTL, H; L OC	0.73
27	37	M	R/L	P	R FC, OC, TC, PC, H; L OC	0.69
28	30	F	R/U	M	R FC, OC, TC, PC	0.66
29	26	F	R/L	T	R FC, TC, PC, MTL, H; L FC, TC, PC, MTL, H	0.77
30	40	F	L/R	J	L FC, TC, PC, MTL, H	0.56
31	35	F	R/L	C	L FC, TC, MTL, H	0.60
32	40	F	R/L	E	R FC, TC; L FC, TC, PC, MTL, H	0.60
33	47	M	R/U	P	R FC, TC, PC, H	0.74
34	38	F	R/U	C	R TC, MTL; L FC, TC, MTL, H	0.44
35	21	F	R/U	M	R FC; L FC, TC	0.76
36	21	M	R/U	M	R FC	0.82
37	25	M	R/L	D	R TC; L FC, OC, TC, PC	0.65
38	52	F	R/L	J	R TC, H; L FC, OC, TC, PC, MTL, H	0.76
39	42	M	R/L	C	R FC; L FC, TC, PC	0.66
40	38	F	R/L	T	R TC, MTL; L FC, OC, TC, PC, MTL, H	0.51
41	52	M	R/L	J	R TC, PC, MTL, H; L TC, PC, MTL, H	0.63
42	38	M	R/L	P	R FC, TC, PC, MTL, H; L FC, TC, MTL, H	0.61
43	38	M	R/L	P	R FC, TC, PC, MTL, H; L FC, TC	0.58
44	32	M	L/L	M	R FC, TC, PC	0.90
45	29	M	R/L	J	L OC, TC, PC, MTL, H	0.84
46	42	M	R/L	J	R FC, OC, TC, PC, MTL, H	0.67
47	29	M	L/L	D	L FC, TC, PC, MTL, H	0.78
48	22	F	R/L	E	R OC, TC, PC, H; L TC, H	0.81
49	23	F	R/L	C	R FC, OC, TC, PC, MTL, H; L PC	0.81
50	30	M	A/U	C	R FC, TC, MTL, H; L FC, TC, PC, MTL, H	0.86

Supplementary Table 1: **H/L**: Handedness/hemispheric language dominance. R: right, L: left, A: ambidextrous, U: undetermined. **Site**: C: Columbia University; D: Dartmouth, E: Emory University; J: Thomas Jefferson University; M: Mayo Clinic; P: Hospital of the University of Pennsylvania; T: University of Texas Southwestern, Dallas. **Electrode locations**: R/L: right/left; FC: frontal cortex; TC: temporal cortex; PC: parietal cortex; OC: occipital cortex; MTL: medial temporal lobe (exclusive of hippocampus); H: hippocampus. **Accuracy**: Mean accuracy of all subject responses, where 0 indicates the worst possible response, 1 indicates a perfect response.

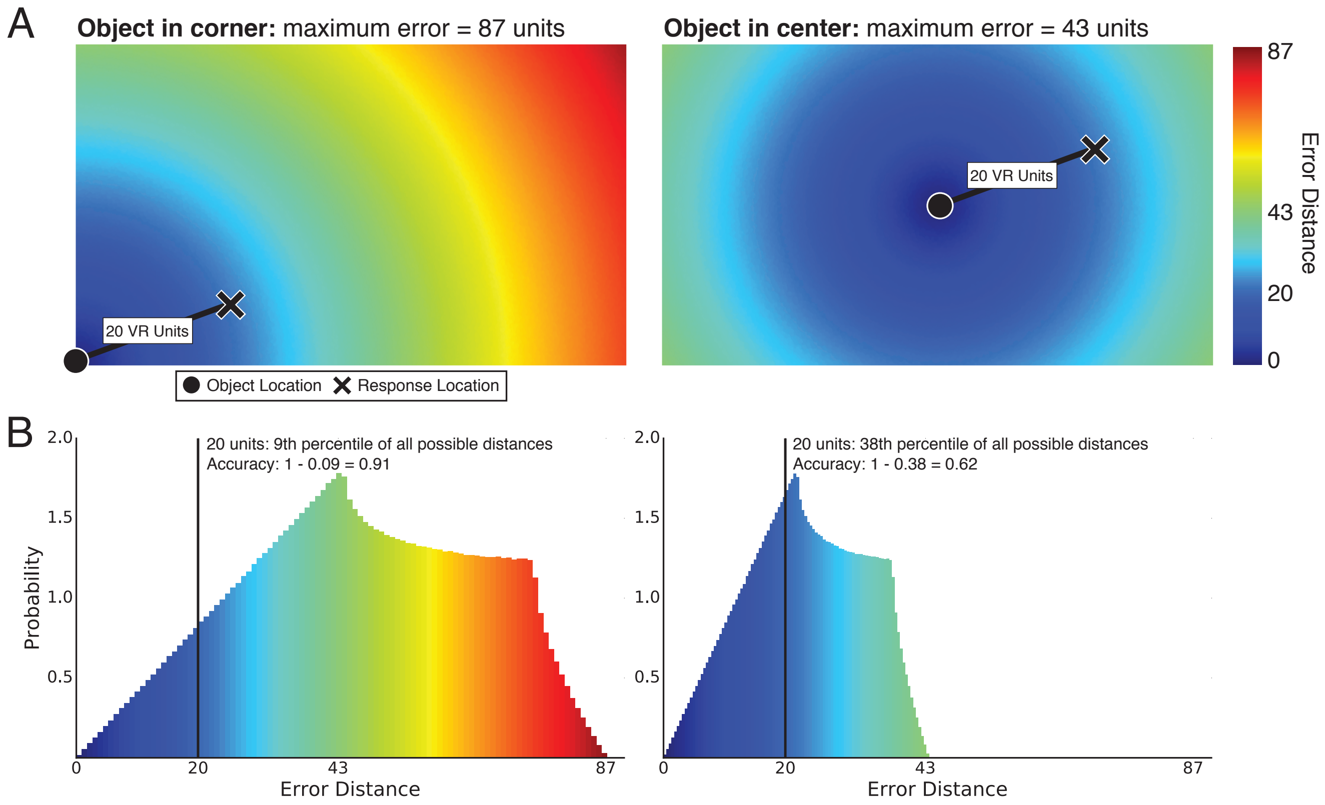

Supplementary Figure 1: **A.** Overhead heat maps showing the possible Euclidean distance errors for an object located in the corner (left) and the center (right) of the environment. **B.** Probability distributions of possible Euclidean errors for the locations shown in A. The vertical line shows where a response of 20 units falls relative to all possible responses.

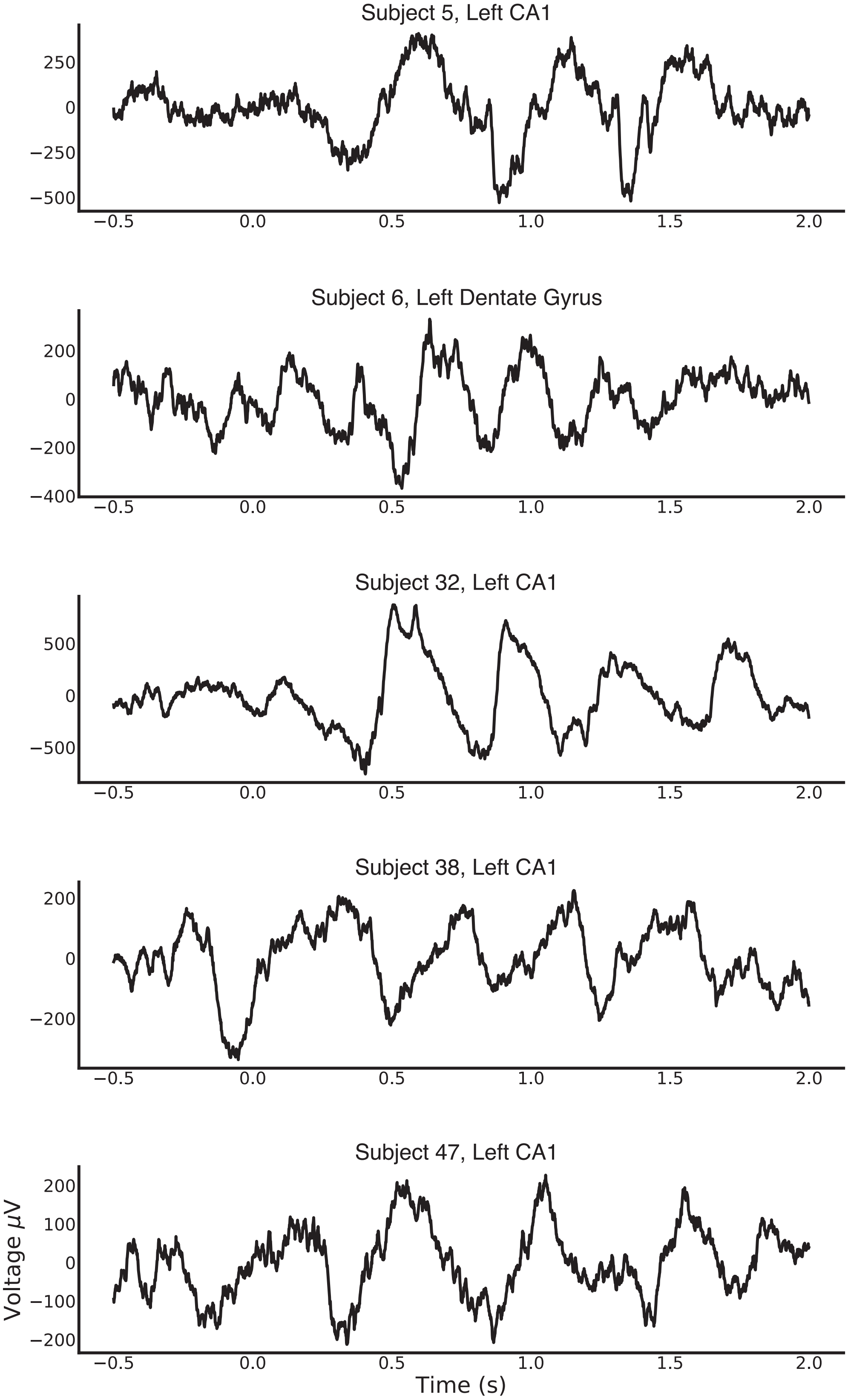

Supplementary Figure 2: Raw hippocampal voltage traces for five successful memory encoding trials, each from a different subject, showing clear low-frequency oscillations during encoding. Traces are unfiltered except for a 60 Hz notch filter to eliminate line noise. The chests opened to reveal an item at 0 s and the item disappeared at 1.5 s.

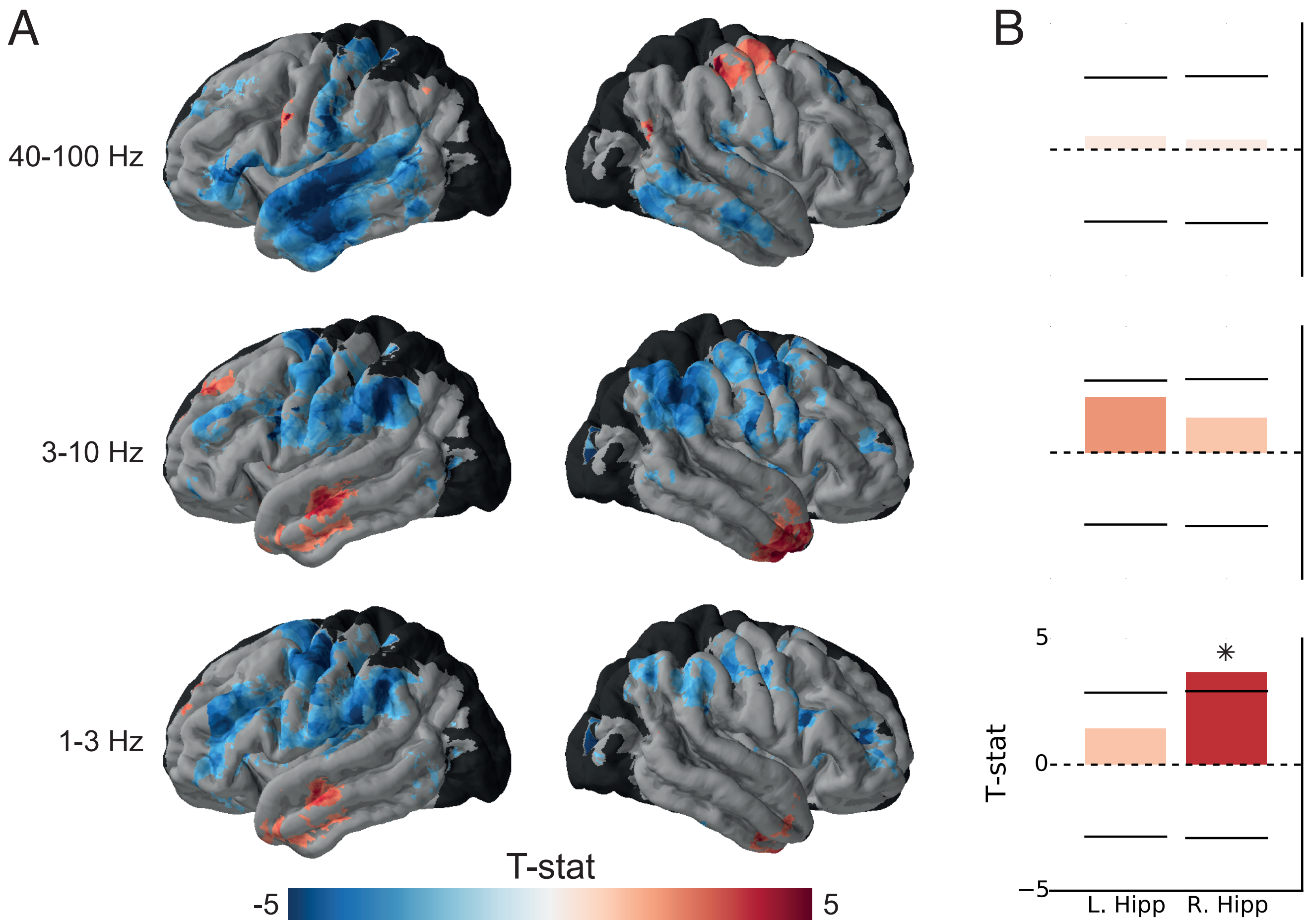

Supplementary Figure 3: Group level navigation-related changes in spectral power in three frequency bands. Red indicates greater power during the navigation condition, blue indicates greater power for the pre-trial baseline condition. Color corresponds to the t-statistic from comparing the distribution of subject-level effects to zero. **A.** Brain plots of significant changes in navigation-related power, thresholded using a permutation procedure (see *Methods*). Non-significant regions are in gray. Regions with less than 5 subjects were not included and are rendered in black. **B.** T-statistics for the left (N=26) and right (N=21) hippocampi. Horizontal bars represent the critical t value for significance, determined by the degrees of freedom of that ROI and a Bonferroni correction.

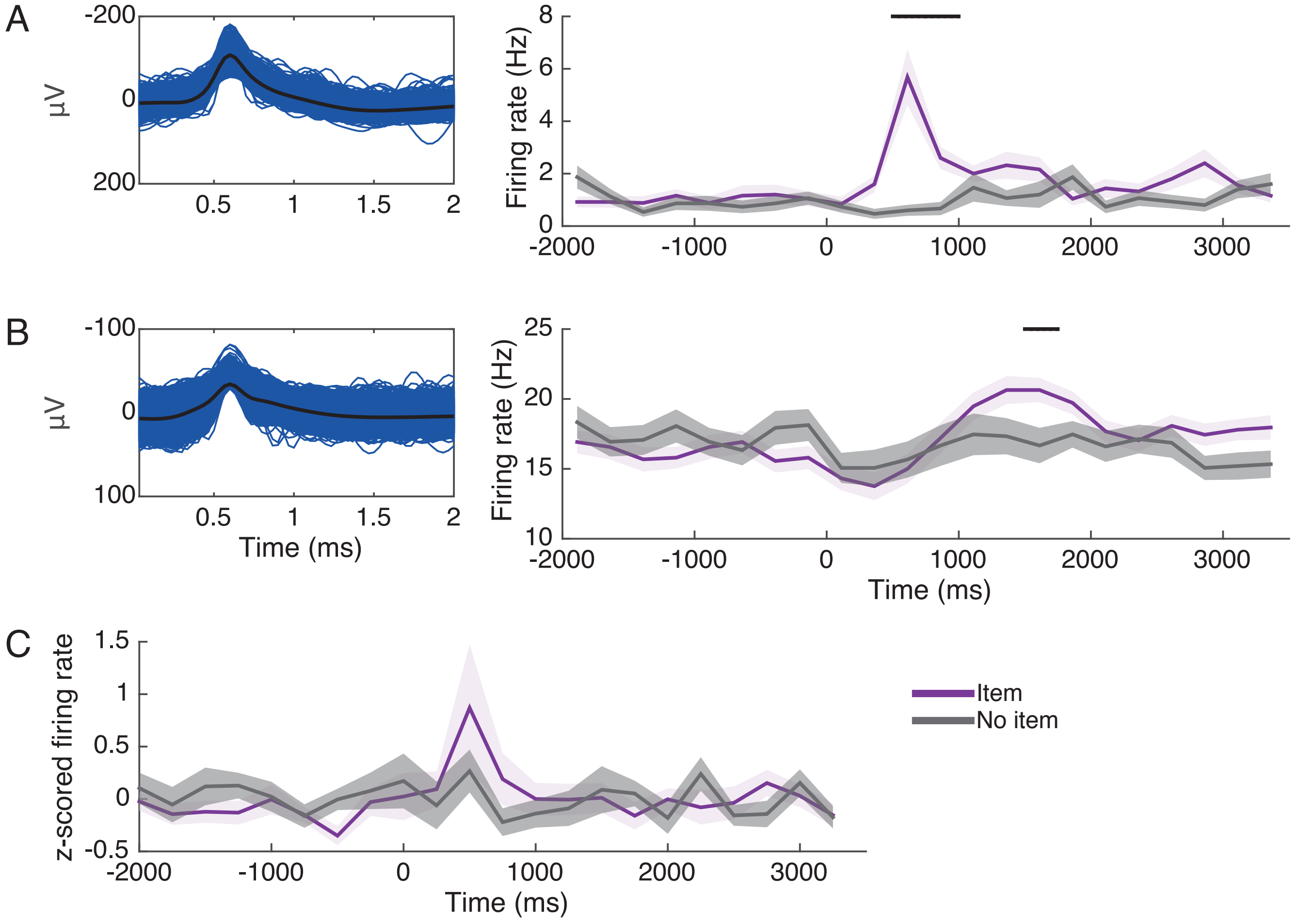

Supplementary Figure 4: **A.** Example neuron in the right hippocampus. Left. The raw waveform of all spikes from this neuron during the task session. Individual waveforms are in blue, the averaged waveform is in black. Right. Averaged firing rate during chest viewing. Firing rate is binned into 250 ms time bins. Error bars indicate ± 1 SEM (n=100 item trials, 60 empty trials). Black bar indicates $p < 0.01$ for a two sample t-test between item and empty trials (d.o.f.=158) for each 250 ms bin. **B.** Example neuron from the left hippocampus of a different subject. Panels follow same format as in A. **C.** Averaged z-scored firing rates from 10 hippocampal neurons, from 4 subjects. Ten neurons out of a total of 18 hippocampal neurons had at least 50 spikes in both conditions and were included in this analysis. Firing rates were z-scored across the time range -2000 - 3500 for each neuron, and the z-scores were then averaged across the neurons. Error bars indicate ± 1 SEM (n=10).

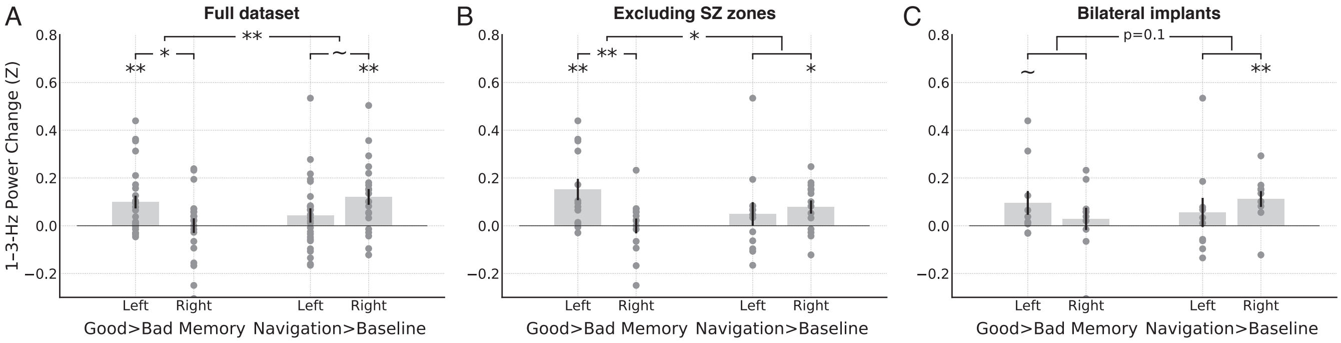

Supplementary Figure 5: Laterality of memory and navigation effects. Task-related 1–3-Hz power changes within left and right hippocampus, as measured by the difference in mean z-scored power, is shown for the recalled/not recalled contrast and the navigation/baseline contrast. Error bars are ±1 SEM. Dots represent individual subjects. $\sim = p < 0.1, * = p < 0.05, ** = p < 0.01$. **A.** Memory and navigations effects based the original dataset, not excluding any epileptic zones (N=26 left, 21 right). **B.** Memory and navigation effects after excluding hippocampi if any ipsilateral medial temporal lobe electrodes were in clinically defined seizure onset zones (N=13 left, 14 right). There was a significant memory effect only in the left hippocampus (left hemisphere t-test: $t(12) = 3.39$, $p < 0.01$, right hemisphere t-test: $t(13) = 0.03$, $p > 0.1$) and a significant navigation effect only in the right hippocampus (left hemisphere t-test: $t(12) = 1.02$, $p > 0.1$, right hemisphere t-test: $t(13) = 2.82$, $p < 0.05$). An ANOVA with factors *hemisphere* (left or right) and *condition* (memory or navigation) still shows significant interaction ($F(1, 50) = 5.5$, $p < 0.05$, **C.** Memory and navigation effects for only patients with bilateral hippocampal implants (N=10).

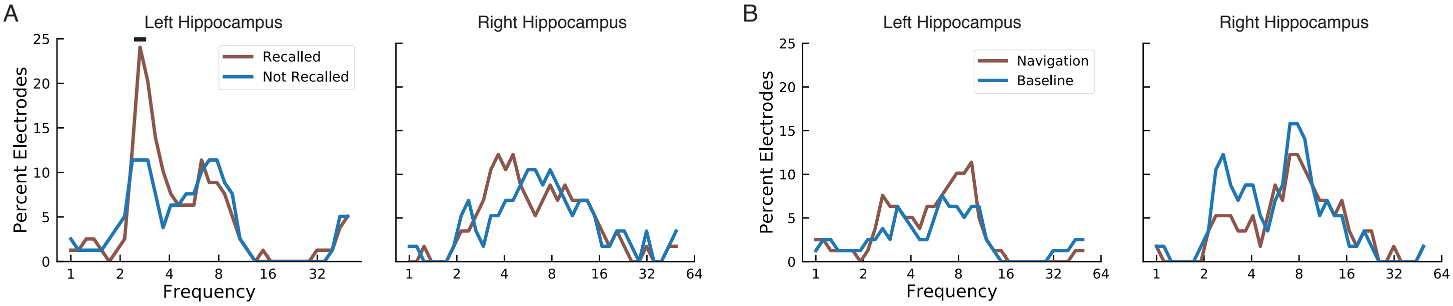

Supplementary Figure 6: The percent of electrodes where oscillations were detected for both left and right hippocampi and for memory (A) and navigation (B) conditions. Black horizontal line indicates a significant ($p < 0.05$ difference between conditions, χ^2 test.)
