# Supplementary material for: Lateralized hippocampal oscillations underlie distinct aspects of human spatial memory and navigation
Source: Nat Commun. 2018 Jun 21;9:2423. doi: 10.1038/s41467-018-04847-9 (PMC6013427; doi:10.1038/s41467-018-04847-9)
Supplement: Supplementary file 3 — Description of Additional Supplementary Files [file 41467_2018_4847_MOESM3_ESM.pdf]

Description of Additional Supplementary Files

File Name: Supplementary Movie 1

Description: Rendering of Patient 1 performing a single trial of the task. Note that the overhead view in the bottom left corner and the frame rate indicator in top left corner were not present during that actual performance of the task. The task was created using the Unity 3D graphics engine.
